# SARS‐CoV‐2 vaccination and infection in ozanimod‐treated participants with relapsing multiple sclerosis

**DOI:** 10.1002/acn3.51862

**Published:** 2023-08-07

**Authors:** Bruce A. C. Cree, Rachel Maddux, Amit Bar‐Or, Hans‐Peter Hartung, Amandeep Kaur, Elizabeth Brown, Yicong Li, Yanhua Hu, James K. Sheffield, Diego Silva, Sarah Harris

**Affiliations:** ^1^ Department of Neurology Weill Institute for Neurosciences, University of California San Francisco San Francisco California USA; ^2^ Bristol Myers Squibb Princeton New Jersey USA; ^3^ Department of Neurology, Center for Neuroinflammation, and Experimental Therapeutics, Perelman School of Medicine University of Pennsylvania Philadelphia Pennsylvania USA; ^4^ Department of Neurology Medical Faculty, Heinrich‐Heine University Düsseldorf Germany; ^5^ Brain and Mind Centre University of Sydney Sydney New South Wales Australia; ^6^ Department of Neurology Medical University of Vienna Vienna Austria; ^7^ Palacký University Olomouc Olomouc Czech Republic

## Abstract

**Objective:**

To investigate the serologic response, predictors of response, and clinical outcomes associated with severe acute respiratory syndrome coronavirus 2 (SARS‐CoV‐2) vaccination and infection in ozanimod‐treated participants with relapsing multiple sclerosis (RMS) from DAYBREAK.

**Methods:**

DAYBREAK (ClinicalTrials.gov‐NCT02576717), an open‐label extension study of oral ozanimod 0.92 mg, enrolled participants aged 18–55 years with RMS who completed phase 1–3 ozanimod trials. Participants who were fully vaccinated against SARS‐CoV‐2 with mRNA or non‐mRNA vaccines, were unvaccinated, and/or had COVID‐19–related adverse events (AEs, with or without vaccination) and postvaccination serum samples were included (*n* = 288). Spike receptor binding domain (RBD) antibody levels (seroconversion: ≥0.8 U/mL) and serologic evidence of SARS‐CoV‐2 infection (nucleocapsid IgG: ≥1 U/mL) were assessed (Roche Elecsys/Cobas e411 platform).

**Results:**

In fully vaccinated participants (*n* = 148), spike RBD antibody seroconversion occurred in 90% (*n* = 98/109) of those without serologic evidence of prior SARS‐CoV‐2 exposure (100% [*n* = 80/80] seroconversion after mRNA vaccination) and in 100% (*n* = 39/39) of participants with serologic evidence of viral exposure. mRNA vaccination predicted higher spike RBD antibody levels, whereas absolute lymphocyte count (ALC), age, body mass index, and sex did not. COVID‐19–related AEs were reported in 10% (*n* = 15/148) of fully vaccinated participants—all were nonserious and not severe; all participants recovered.

**Interpretation:**

Most ozanimod‐treated participants with RMS mounted a serologic response to SARS‐CoV‐2 vaccination and infection, regardless of participant characteristics or ALC levels. In this analysis, all COVID‐19–related AEs post–full vaccination in participants taking ozanimod were nonserious and not severe.

## Introduction

Ozanimod, a sphingosine 1‐phosphate (S1P) receptor modulator that binds with high affinity selectively to S1P receptor subtypes 1 and 5, is approved in multiple countries for the treatment of adults with either relapsing forms of multiple sclerosis (RMS) or moderately to severely active ulcerative colitis.^1^, ^2^ Ozanimod blocks lymphocyte egress from lymphoid tissue, reducing the number of lymphocytes in peripheral blood, and is hypothesized to exert therapeutic effects in RMS by limiting lymphocyte migration into the central nervous system.^1^, ^2^, ^3^

In an exploratory analysis of a phase 1 study, ozanimod reduced absolute lymphocyte count (ALC) levels in the peripheral blood of participants with RMS, an effect that was primarily driven by decreases in circulating T and B cells, with minimal effects on levels of other leukocyte types (e.g., monocytes, natural killer cells, and natural killer T cells).^4^ T and B lymphocytes play a role in the production of antibodies and immunological memory against severe acute respiratory syndrome coronavirus 2 (SARS‐CoV‐2)^5^; therefore, multiple sclerosis (MS) disease‐modifying therapies (DMTs) that sequester (e.g., S1P receptor modulators) or deplete (e.g., anti‐CD20 therapies) T and/or B cells may impact the serologic response to SARS‐CoV‐2 vaccination or infection.^5^, ^6^, ^7^, ^8^, ^9^, ^10^, ^11^, ^12^, ^13^, ^14^, ^15^

The ability of ozanimod‐treated participants to mount a response to SARS‐CoV‐2 infection was observed in an interim analysis of the DAYBREAK open‐label extension trial, where COVID‐19–related adverse events (AEs) were largely nonserious, and most participants recovered without sequelae.^16^ There is insufficient evidence available regarding the impact of ozanimod on the serologic response to SARS‐CoV‐2 vaccination and/or infection. Given that most patients with MS are willing to be vaccinated against SARS‐CoV‐2,^17^ the primary aim of this retrospective analysis was to investigate the serologic response to SARS‐CoV‐2 vaccination and clinical outcomes of COVID‐19 in vaccinated ozanimod‐treated participants with RMS from the DAYBREAK trial, with a secondary aim of determining the serologic response to infection.

## Methods

### Study design

Participants with RMS who completed any of the four ozanimod phase 1, 2, or 3 “parent” trials were eligible to enter a single‐arm, open‐label, phase 3 trial of oral ozanimod 0.92 mg (DAYBREAK, ClinicalTrials.gov: NCT02576717; EudraCT: 2015‐002500‐91).^16^ The phase 1 trial was a randomized, 12‐week, open‐label pharmacokinetic/pharmacodynamic study of oral ozanimod 0.46 or 0.92 mg/d.^4^ The phase 2 study was a randomized, double‐blind, 24‐week, placebo‐controlled study of oral ozanimod 0.46 or 0.92 mg/d, followed by a 24‐month dose‐blinded extension where all participants received ozanimod.^18^ The two phase 3 trials (RADIANCE and SUNBEAM) were randomized, double‐blind trials comparing oral ozanimod 0.46 or 0.92 mg/d with intramuscular interferon β‐1a 30 μg/wk—phase 3 RADIANCE lasted 24 months and SUNBEAM continued until the last participant was treated for 12 months.^19^, ^20^ Participants from the phase 3 trials underwent a 1‐week dose escalation upon entry into DAYBREAK (participants received ozanimod 0.23 mg on days 1–4, ozanimod 0.46 mg on days 5–7, and then their assigned dose of ozanimod 0.92 mg on day 8 and thereafter), whereas dose escalation was not performed for participants from the other trials unless there was more than a 14‐day gap in treatment. DAYBREAK began on October 16, 2015, and all parent trials were completed by October 20, 2017; thus, all participants were receiving ozanimod 0.92 mg prior to the COVID‐19 pandemic. DAYBREAK was conducted in 25 countries in Europe and North America, plus South Africa and New Zealand, and was completed in January 2023.

The phase 1, 2, and 3 studies were approved by institutional review boards and were designed and monitored in compliance with the principles of Good Clinical Practice as required by regulatory authorities and in accordance with the Declaration of Helsinki. All participants provided written informed consent, and all DAYBREAK participants reconsented.

### Participants

Upon entry into the phase 1–3 studies, participants were 18–55 years of age with RMS, had brain magnetic resonance imaging lesions consistent with MS, and had an Expanded Disability Status Scale score of 0–5.0 (phases 2 and 3) or 0–6.0 (phase 1). Participants in DAYBREAK who were fully vaccinated by October 11, 2021, and/or had COVID‐19–related AEs (with or without vaccination) and serum samples available postvaccination were included in this analysis (*n* = 148). In this set of participants, serum samples were available at time points ranging from 1 day to 229 days postvaccination. All unvaccinated participants in the serologic dataset (*n* = 209) had investigator reported COVID‐19–related AEs before October 11, 2021; however, some (*n* = 69) were not included in the analysis because they were not detected to have nucleocapsid antibodies. The remaining unvaccinated participants with detectable nucleocapsid antibodies (*n* = 140) were included.

### Procedures

Serum samples were collected every 3 months throughout DAYBREAK, until February 2020, when the protocol was amended to include measurements every 3 months for 3 years and every 6 months thereafter. Participants in DAYBREAK received vaccination against SARS‐CoV‐2 at their, and the investigator's, discretion. In this analysis, participants were grouped by mRNA (Moderna or Pfizer) or non‐mRNA (AstraZeneca, Johnson & Johnson, Sinopharm, or Sputnik) vaccine types, as mRNA vaccines have been associated with increased seroconversion rates compared with non‐mRNA vaccines in patients with MS.^21^ *Fully vaccinated* was defined as two doses of vaccine, except for Johnson & Johnson where one dose was considered fully vaccinated. Serology outcomes were assessed from January 13, 2020, to October 11, 2021; clinical outcomes were assessed through January 28, 2022. A wider window was used for clinical outcomes to allow for more outcomes to be observed after vaccination.

### Serology analysis

The Elecsys anti‐SARS‐CoV‐2 assay and Cobas e411 analyzer (F. Hoffmann‐La Roche, Basel, Switzerland) were used, according to the manufacturer's instructions, to measure SARS‐CoV‐2 spike protein receptor binding domain (RBD) antibodies and SARS‐CoV‐2 nucleocapsid antibodies.^22^ The anti‐SARS‐CoV‐2 assay detects the presence of low levels of spike RBD antibodies with high sensitivity (97.92%; 95% confidence interval: 95.21–99.32) and specificity (99.95%; 95% confidence interval: 99.87–99.99).^23^ Per the manufacturer's instructions, spike RBD antibody levels ≥0.8 U/mL were considered an indicator of seroconversion (i.e., the lowest quantity of antibody that determines reactivity for spike RBD‐specific antibodies),^24^ and nucleocapsid antibody seroconversion was defined as immunoglobulin G (IgG) levels ≥1 U/mL (i.e., seropositivity due to SARS‐CoV‐2 infection).^25^ Participants who were nucleocapsid antibody negative are referred to as those who did not have serologic evidence of SARS‐CoV‐2 exposure, and participants who were nucleocapsid antibody positive are referred to as those who had serologic evidence of SARS‐CoV‐2 exposure.

For COVID‐19–related AEs, cases were considered confirmed by polymerase chain reaction or antigen testing. Suspected COVID‐19 cases were pending confirmation of SARS‐CoV‐2 infection from the site, had a negative polymerase chain reaction test, or were considered as such at the investigator's discretion.

### AE reporting

COVID‐19–related AEs were reported and characterized by investigators in terms of severity and seriousness at their discretion. An event was considered mild if it was transient and did not interfere with the participant's daily activities, moderate if it introduced a low level of inconvenience or concern to the participant and may have interfered with daily activities, or severe if it was incapacitating and interrupted the participant's usual daily activity. An AE was considered serious if it was life‐threatening, required hospitalization or prolongation of existing hospitalization, or resulted in significant disability, a congenital abnormality, or death.

### Statistical analyses

Descriptive statistics for the different exposure and vaccination status groups were provided based on the distribution of the reported variable; spike RBD antibody levels were log‐transformed for data visualization (log10 was used to expedite comparisons with existing published data) and analysis (log2 was used as a normalizing data transformation). Differences (*p* value <0.05) between prevaccinated/unvaccinated participants and vaccinated participants, non‐mRNA and mRNA vaccine groups, and participants with and without serologic evidence of SARS‐CoV‐2 exposure were tested via unpaired two‐tailed *t*‐test for log2 SARS‐CoV‐2 spike RBD antibody levels. A linear model regressed log2 SARS‐CoV‐2 spike RBD levels on vaccine type (including none), age, log2‐ALC, body mass index (BMI), and sex to determine predictors of spike RBD antibody levels (in participants with and without serologic evidence of SARS‐CoV‐2 exposure) and seroconversion (in participants with serologic evidence of SARS‐CoV‐2 exposure). A Fisher exact test was used to test the association of seroconversion with vaccine type in participants without serologic evidence of SARS‐CoV‐2 exposure. Due to the retrospective, exploratory nature of this study, all statistical comparisons are considered hypothesis generating rather than declarative, and *p* values are nominal.

## Results

### Participant disposition and baseline characteristics

**Figure 1:**
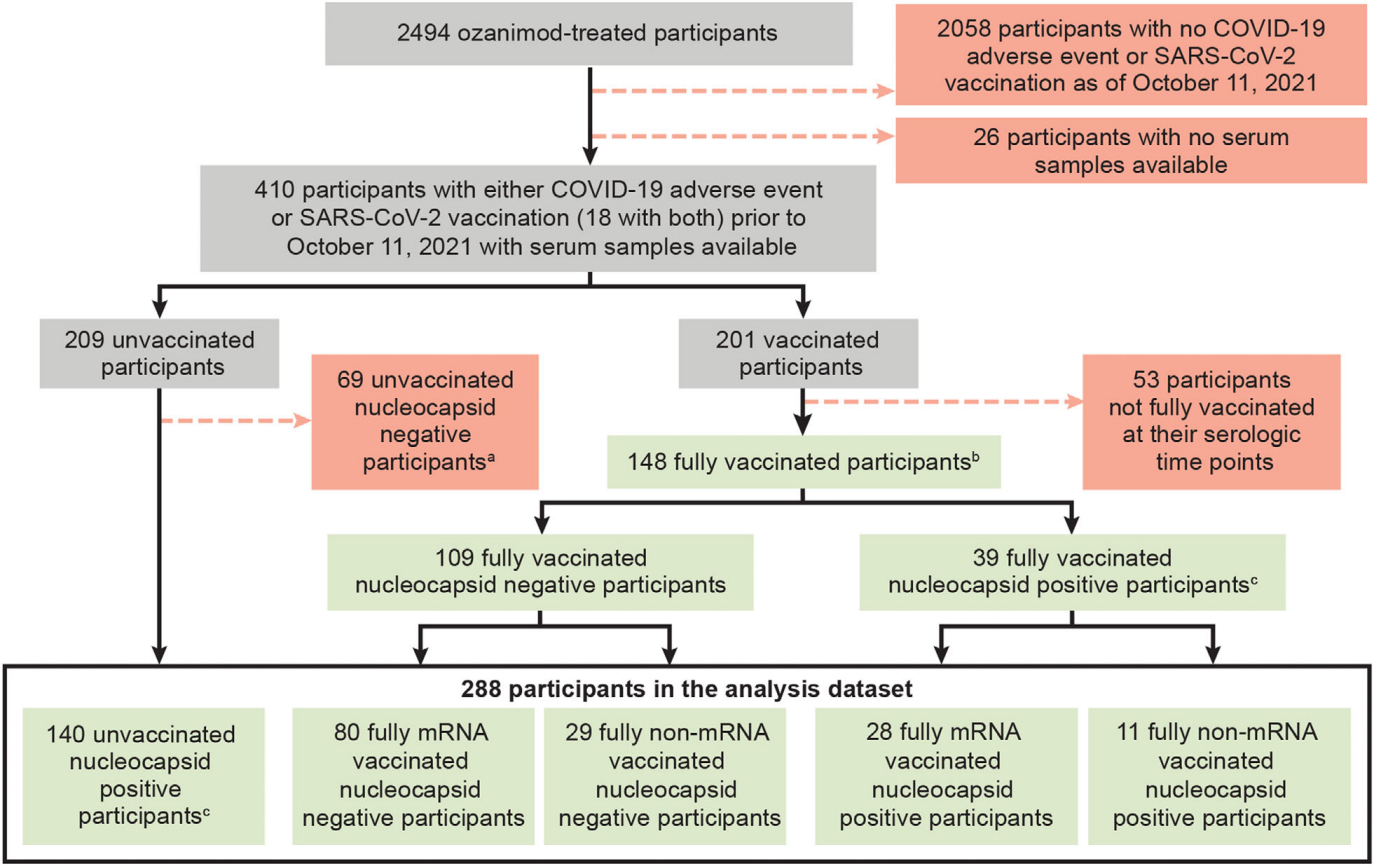
Flow diagram of participants included in this analysis. ^a^All unvaccinated participants in the serologic dataset had COVID‐19 adverse events prior to October 11, 2021; however, some were not included in the analysis because they were not detected to have nucleocapsid antibodies. COVID‐19 may have only been suspected, or in some cases the adverse event preceded the serologic time points by many months. ^b^Fully vaccinated prior to at least 1 serologic time point. ^c^SARS‐CoV‐2 nucleocapsid antibodies were detected for at least 1 serologic time point. Participants who were nucleocapsid antibody negative did not have serologic evidence of SARS‐CoV‐2 exposure, and participants who were nucleocapsid antibody positive had serologic evidence of SARS‐CoV‐2 exposure. COVID‐19, coronavirus disease 2019; SARS‐CoV‐2, severe acute respiratory syndrome coronavirus 2.

Among the 288 participants with serologic data from DAYBREAK (January 2020–October 2021), 109 were nucleocapsid antibody negative (no serologic evidence of SARS‐CoV‐2 exposure) and 179 were nucleocapsid antibody positive (serologic evidence of SARS‐CoV‐2 exposure; Fig. 1). Of the 288 participants with serologic data included in this analysis, 148 were fully vaccinated with either mRNA (Moderna or Pfizer; *n* = 108) or non‐mRNA (AstraZeneca, Johnson & Johnson, Sinopharm, or Sputnik; *n* = 40) vaccines, and 140 participants were not vaccinated (Fig. 1). Baseline demographics and disease characteristics were relatively similar between the vaccinated and unvaccinated groups, except unvaccinated participants were younger (mean age of 36.7 years) than vaccinated participants (mean age of 41.5 years, *p* < 0.0001; Table 1). Mean (range) exposure to ozanimod was 2120 (1372–3066) days for the 288 participants included in this analysis.

**Table 1:** Baseline demographics and characteristics of vaccinated and unvaccinated participants.

	Analysis population (*n* = 288)		
	Vaccinated (*n* = 148)		Unvaccinated (*n* = 140)
	Nucleocapsid antibody positive (*n* = 39)	Nucleocapsid antibody negative (*n* = 109)	Nucleocapsid antibody positive (*n* = 140)
Spike RBD antibody seroconversion, *n* %			
Positive	39 (100)	98 (90)	135 (96)
Negative	0	11 (10)	5 (4)
Age, years (range)	44 (24–56)	41 (23–56)	37 (19–56)
Female, *n* %	29 (74)	80 (73)	95 (68)
ALC **×** 10^9^/L^a^ (range)	0.9 (0.3–2.9)	0.7 (0.2–2.2)	0.7 (0.1–3.0)
BMI, kg/m^2^ (range)	25 (13–39)	25 (17–42)	25 (14–45)
Total ozanimod exposure, days (range)	2103 (1455–2823)	2177 (1448–3066)	2080 (1372–2948)

Spike RBD antibody levels ≥0.8 U/mL were considered an indicator of seroconversion. Participants who were nucleocapsid antibody negative did not have serologic evidence of SARS‐CoV‐2 exposure, and participants who were nucleocapsid antibody positive had serologic evidence of SARS‐CoV‐2 exposure.

ALC, absolute lymphocyte count; BMI, body mass index; RBD, receptor binding domain; SARS‐CoV‐2, severe acute respiratory syndrome coronavirus 2.

^a^ Normal range: 1.0–3.4 × 10^9^/L.

### Response to SARS‐CoV‐2 vaccination in participants without prior SARS‐CoV‐2 exposure

**Figure 2:**
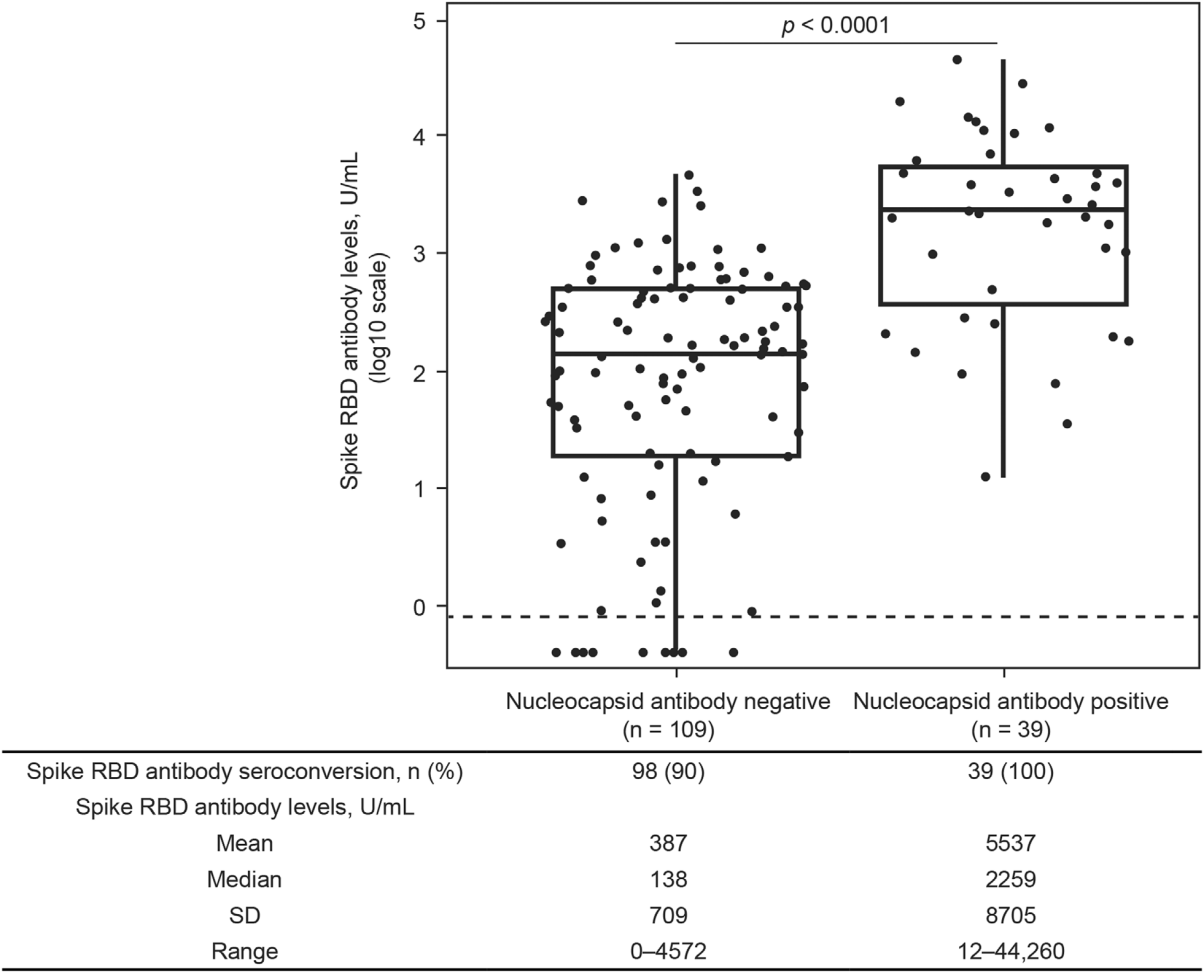
Serologic response to SARS‐CoV‐2 vaccination. Spike RBD antibody levels ≥0.8 U/mL were considered an indicator of seroconversion. The horizontal dashed line represents the seroconversion threshold (0.8 U/mL). Participants who were nucleocapsid antibody negative did not have serologic evidence of SARS‐CoV‐2 exposure, and participants who were nucleocapsid antibody positive had serologic evidence of SARS‐CoV‐2 exposure. The *p* value was determined via *t*‐test for log2 SARS‐CoV‐2 spike RBD antibody levels. RBD, receptor binding domain; SARS‐CoV‐2, severe acute respiratory syndrome coronavirus 2; SD, standard deviation.

**Figure 3:**
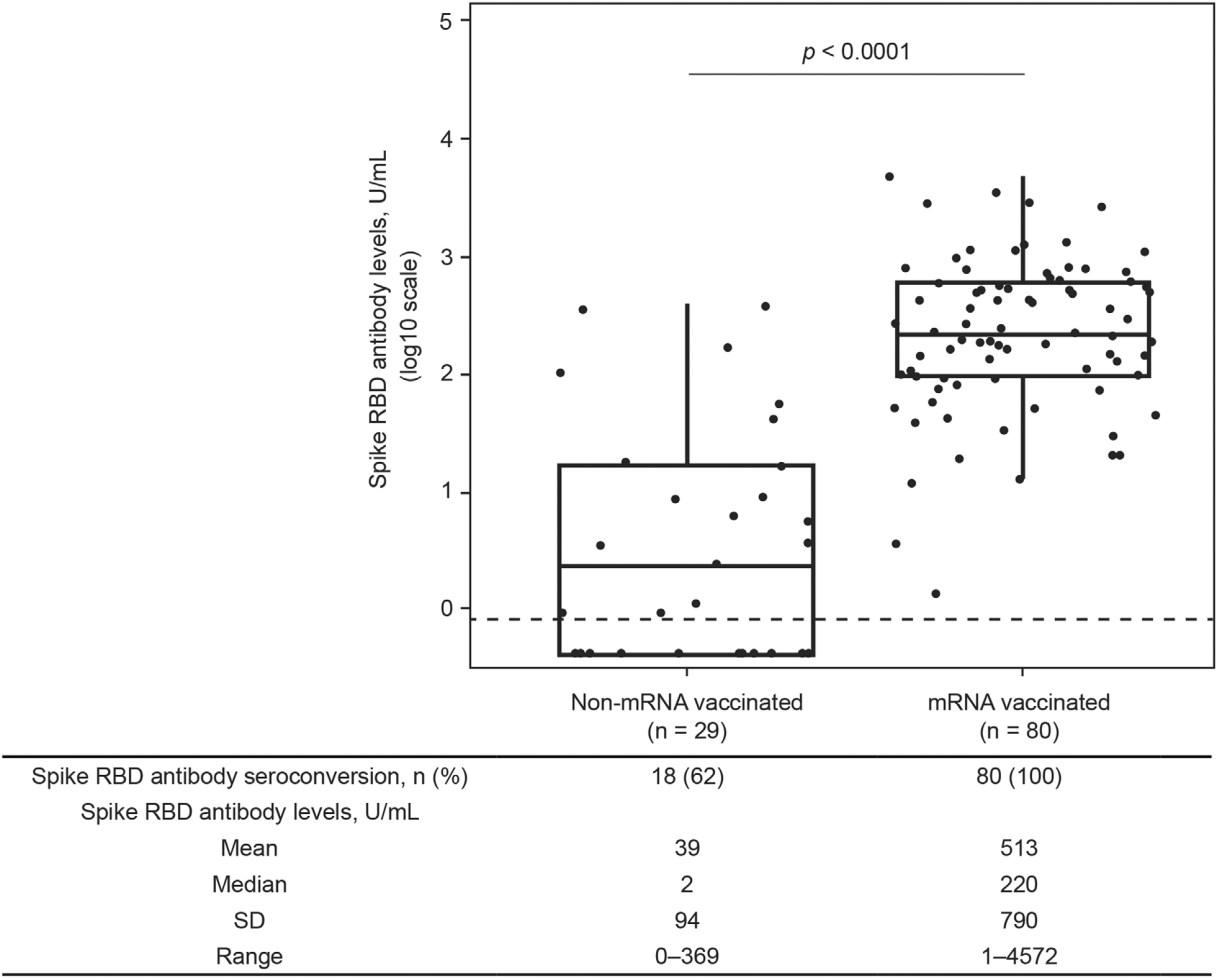
Serologic response to non‐mRNA and mRNA SARS‐CoV‐2 vaccinations in participants with no serologic evidence of SARS‐CoV‐2 exposure. Spike RBD antibody levels ≥0.8 U/mL were considered an indicator of seroconversion. The horizontal dashed line represents the seroconversion threshold (0.8 U/mL). Mean pre‐vaccination spike RBD antibody levels for each group were below the lower limit of quantitation of the assay (0.4 U/mL). Participants who were nucleocapsid antibody negative did not have serologic evidence of SARS‐CoV‐2 exposure. The *p* value was determined via *t*‐test for log2 SARS‐CoV‐2 spike RBD antibody levels. RBD, receptor binding domain; SARS‐CoV‐2, severe acute respiratory syndrome coronavirus 2; SD, standard deviation.

Samples used to measure spike RBD antibody indices were collected a median (interquartile range) of 60 (32–85) days postvaccination. Spike RBD antibody seroconversion (≥0.8 U/mL spike RBD antibody levels) occurred in 90% (98/109) of fully vaccinated participants with no serologic evidence of SARS‐CoV‐2 exposure, with the group having a spike RBD antibody mean (range) of 386.7 (0.4–4572) U/mL (Fig. 2). In fully vaccinated participants with no serologic evidence of SARS‐CoV‐2 exposure, significantly higher spike RBD antibody levels were observed with mRNA versus non‐mRNA vaccines (mean [range]: 512.6 [1.3–4572] U/mL versus 39.3 [0.4–368.5] U/mL, respectively, *p* < 0.0001; Fig. 3). A total of 100% (80/80) of participants who received an mRNA vaccine seroconverted, whereas seroconversion occurred in 62% (18/29) of participants receiving a non‐mRNA vaccine (Fig. 3). All participants who received the AstraZeneca vaccine seroconverted (n = 5/5). Participants who did not seroconvert (11/29) received either the Johnson & Johnson (*n* = 3/6), Sinopharm (*n* = 6/16), or Sputnik (*n* = 2/2) vaccines.

**Figure 4:**
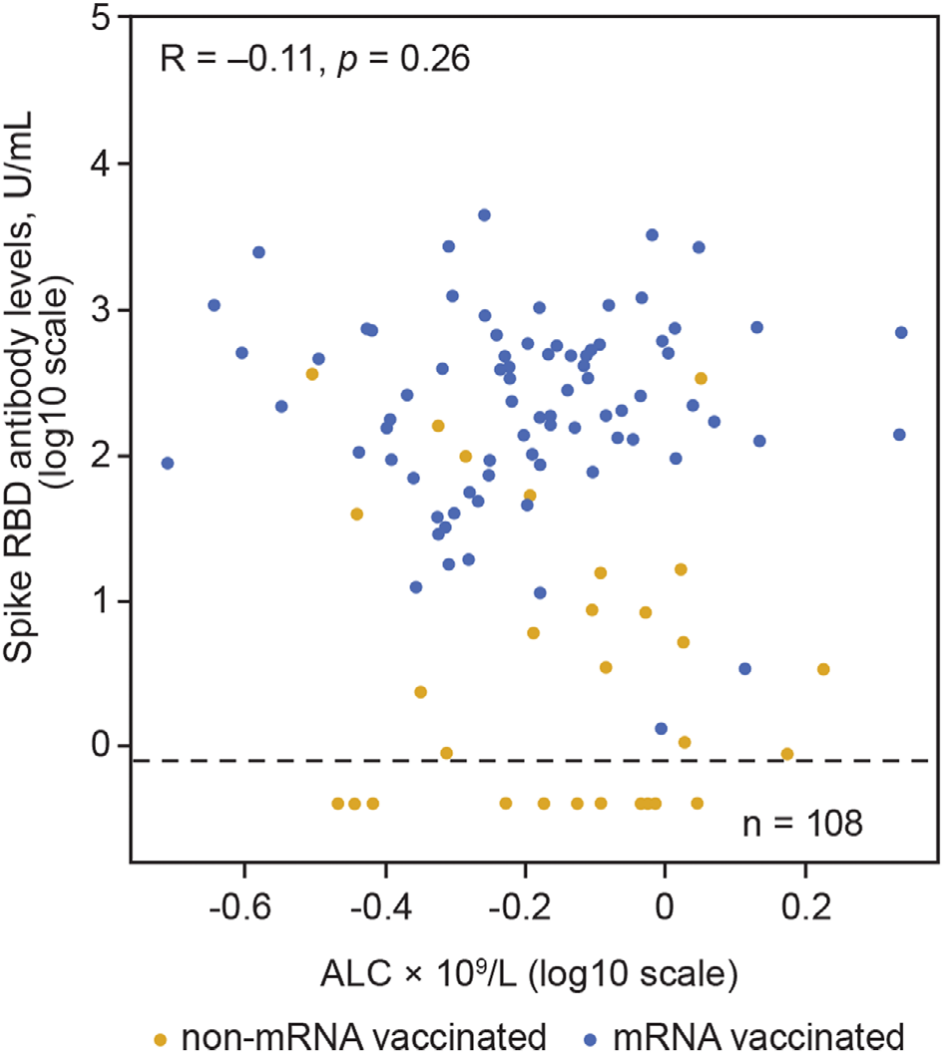
Association between ALC and spike RBD antibody levels in participants with no serologic evidence of SARS‐CoV‐2 exposure. Spike RBD antibody levels ≥0.8 U/mL were considered an indicator of seroconversion. The horizontal dashed line represents the seroconversion threshold (0.8 U/mL). Participants who were nucleocapsid antibody negative did not have serologic evidence of SARS‐CoV‐2 exposure. A linear model regressed log2 SARS‐CoV‐2 spike RBD antibody levels on vaccine type and log2 ALC levels; age, body mass index, and sex were controlled for in the model. ALC, absolute lymphocyte count; RBD, receptor binding domain; SARS‐CoV‐2, severe acute respiratory syndrome coronavirus 2.

In 108 fully vaccinated participants with no serologic evidence of SARS‐CoV‐2 exposure for whom postvaccination samples were available, mRNA vaccines were predictive of higher antibody levels (linear regression: 60‐fold increase) and seroconversion (Fischer exact: *p* < 0.0001) versus non‐mRNA vaccines. There was no evidence of an association between participant characteristics and serologic response to SARS‐CoV‐2 vaccination. Age, ALC, BMI, and sex were not predictive of spike RBD antibody levels in fully vaccinated participants without serologic evidence of SARS‐CoV‐2 exposure (ALC is shown in Fig. 4).

### Response to SARS‐CoV‐2 vaccination in participants with prior SARS‐CoV‐2 exposure

Spike RBD antibody seroconversion occurred in 100% (39/39) of fully vaccinated participants with serologic evidence of SARS‐CoV‐2 exposure (Fig. 2). Significantly higher spike RBD antibody levels were observed in fully vaccinated participants with serologic evidence of SARS‐CoV‐2 exposure compared with those without exposure (mean [range]: 5537 [12.4–44,260] U/mL versus 386.7 [0.4–4572] U/mL, respectively, *p* < 0.0001; Fig. 2). Among participants who had serologic evidence of SARS‐CoV‐2 exposure after vaccination, 67% (26/39) were nucleocapsid antibody positive before vaccination, and 3% (1/39) were nucleocapsid antibody positive after vaccination. For the remaining 31% (12/39), nucleocapsid antibodies were detected in the first sample following vaccination; therefore, we were unable to determine whether vaccination or SARS‐CoV‐2 exposure occurred first. Samples for those 12 participants go directly from prevaccination/pre‐exposure to postvaccination/postexposure, and only the vaccination date is definitively known.

**Figure 5:**
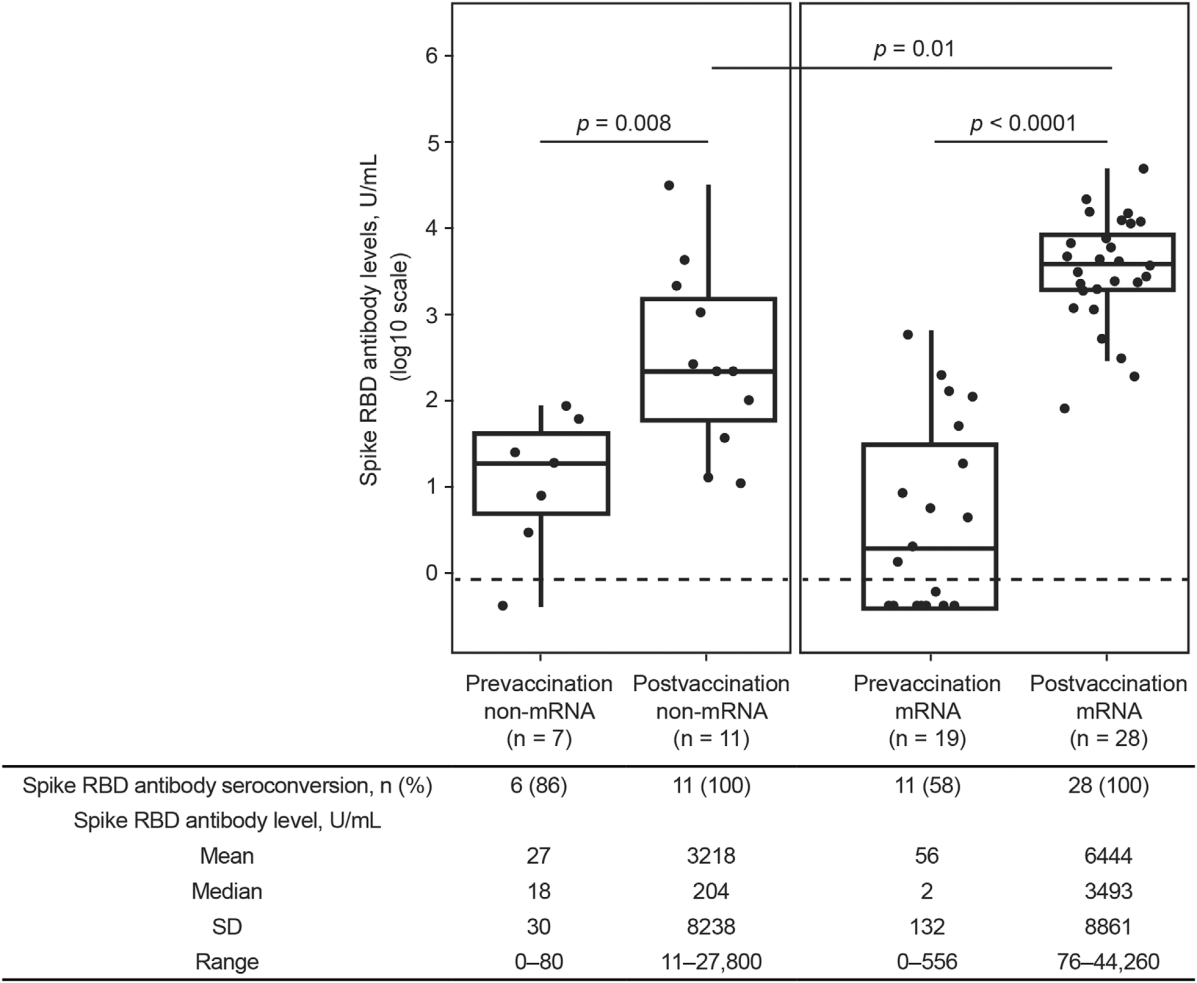
Serologic response to non‐mRNA and mRNA SARS‐CoV‐2 vaccinations in participants with serologic evidence of SARS‐CoV‐2 exposure. Spike RBD antibody levels ≥0.8 U/mL were considered an indicator of seroconversion. The horizontal dashed line represents the seroconversion threshold (0.8 U/mL). Participants who were nucleocapsid antibody positive had serologic evidence of SARS‐CoV‐2 exposure. The *p* values were determined via *t*‐test for log2 SARS‐CoV‐2 spike RBD antibody levels. RBD, receptor binding domain; SARS‐CoV‐2, severe acute respiratory syndrome coronavirus 2; SD, standard deviation.

In participants with serologic evidence of SARS‐CoV‐2 exposure, significantly higher spike RBD antibody levels were observed after vaccination, with mean (range) levels increasing from 27 (0.4–80.2) U/mL prevaccination to 3218 (10.7–27,800) U/mL after non‐mRNA vaccination (*p* = 0.008) and from 55.6 (0.4–555.5) U/mL prevaccination to 6444 (76.2–44,260) U/mL after mRNA vaccination (*p* < 0.0001; Fig. 5). Spike RBD antibody levels were significantly higher in those who received an mRNA vaccine compared with those who received a non‐mRNA vaccine (mean [range]: 6444 [76.2–44,260] U/mL versus 3218 [10.7–27,800] U/mL, respectively, *p* = 0.01; Fig. 5). Seroconversion occurred in 100% (39/39) of fully vaccinated participants, regardless of vaccination type (Fig. 5). In the 179 participants with serologic evidence of SARS‐CoV‐2 exposure, mRNA vaccines were predictive of higher antibody levels (linear regression adjusted for age, BMI, and sex: 8.8‐fold increase in levels) compared with non‐mRNA vaccines. Age, BMI, and sex were not predictive of spike RBD antibody levels after exposure to SARS‐CoV‐2.

### Longitudinal serologic response to SARS‐CoV‐2 exposure and SARS‐CoV‐2 vaccination

**Figure 6:**
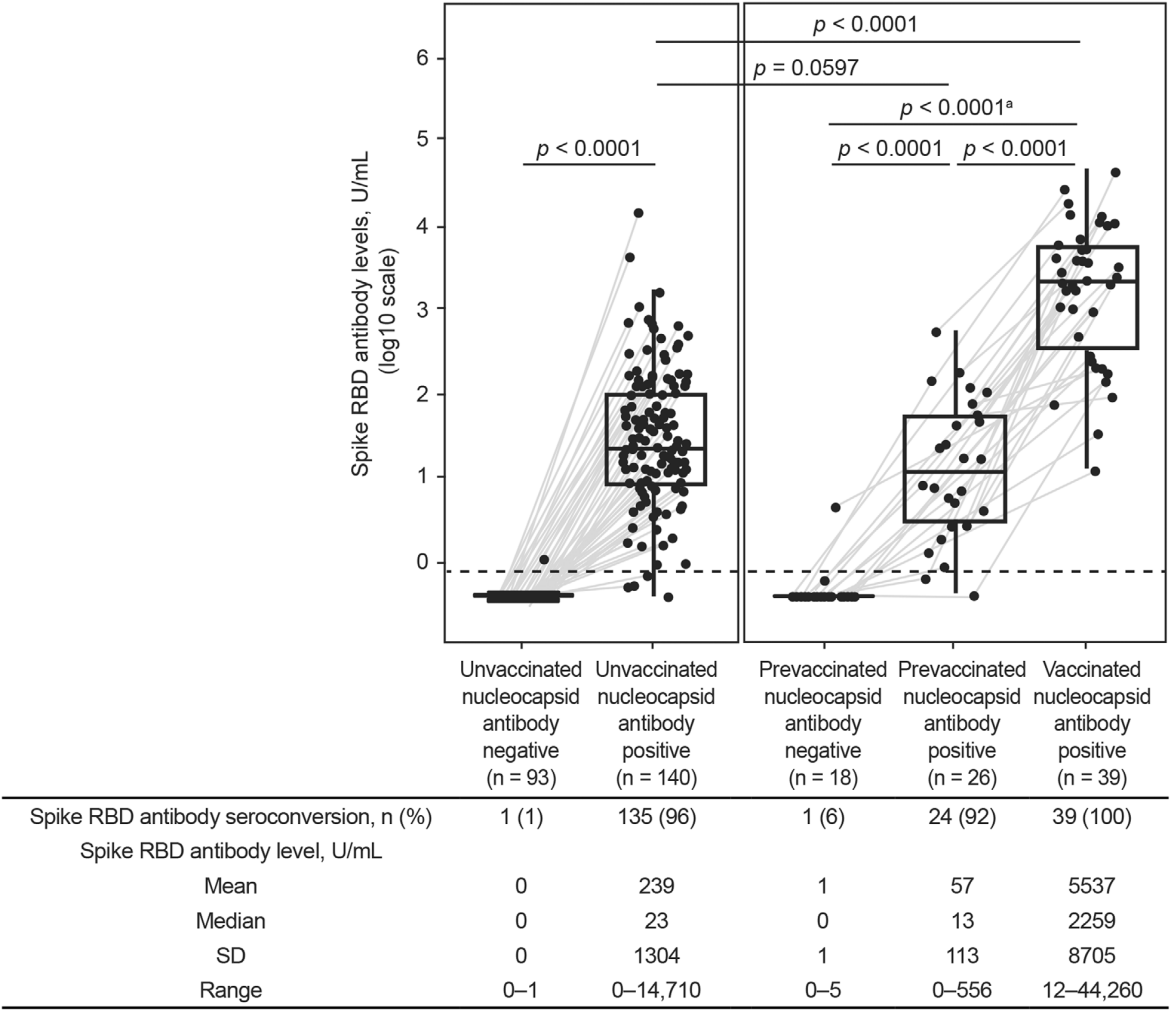
Longitudinal serologic response to SARS‐CoV‐2 vaccination and infection. ^a^Nine of 18 participants had serologic data available before SARS‐CoV‐2 exposure prior to vaccination; *p* < 0.0001 for the whole analysis set and the 9/18 participants with serologic data available before SARS‐CoV‐2 exposure prior to vaccination. Spike RBD antibody levels ≥0.8 U/mL were considered an indicator of seroconversion. The horizontal dashed line represents the seroconversion threshold (0.8 U/mL). Participants who were nucleocapsid antibody negative did not have serologic evidence of SARS‐CoV‐2 exposure, and participants who were nucleocapsid antibody positive had serologic evidence of SARS‐CoV‐2 exposure. Gray lines connect data from serum samples collected from the same participant before and after SARS‐CoV‐2 vaccination and/or exposure. Sample sizes varied due to the retrospective nature of the study, and participants were not matched before or after SARS‐CoV‐2 vaccination and/or exposure due to these sample size limitations. A total of 93/140 unvaccinated participants had samples available pre‐exposure, 26/39 vaccinated nucleocapsid positive participants had samples available prevaccination but postexposure, and 18/39 participants had samples available prevaccination and pre‐exposure. The *p* values were determined by comparing log2 SARS‐CoV‐2 spike RBD antibody level differences between groups using unpaired *t*‐tests. RBD, receptor binding domain; SARS‐CoV‐2, severe acute respiratory syndrome coronavirus 2; SD, standard deviation.

Unvaccinated participants with serologic evidence of SARS‐CoV‐2 exposure mounted a spike RBD antibody response (mean [range]: 238.6 [0.4–14,710 U/mL]; Fig. 6). In a subset of participants with serologic data available before SARS‐CoV‐2 exposure prior to vaccination (*n* = 9/18), spike RBD antibody levels rose after exposure and rose further following full vaccination (*p* < 0.0001; Fig. 6). In participants with serologic evidence of SARS‐CoV‐2 exposure, those who were vaccinated had higher spike RBD antibody levels than those who were unvaccinated (mean [range]: 5537 [12.4–44,260] U/mL versus 238.6 [0.4–14,710] U/mL, respectively, *p* < 0.0001; Fig. 6).

**Figure 7:**
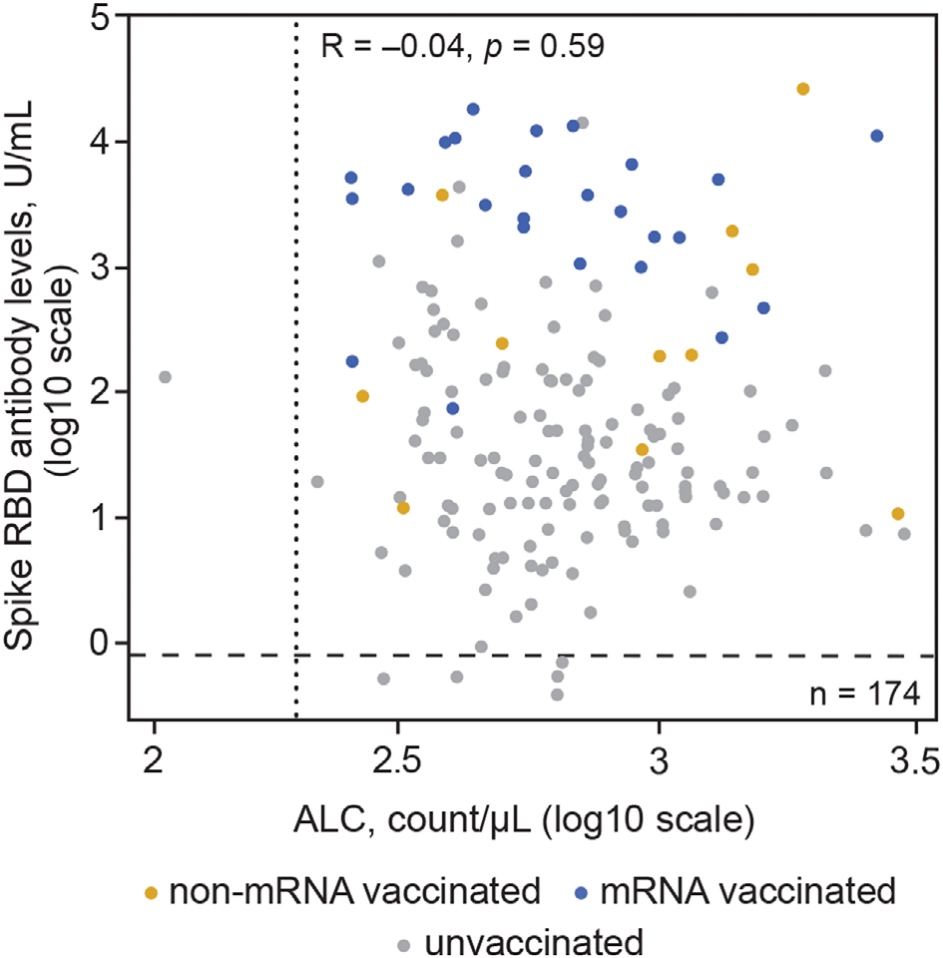
Association between ALC and spike RBD antibody levels in participants with serologic evidence of SARS‐CoV‐2 exposure with and without SARS‐CoV‐2 vaccination. Spike RBD antibody levels ≥0.8 U/mL were considered an indicator of seroconversion. The horizontal dashed line represents the seroconversion threshold (0.8 U/mL), and the vertical dotted line represents 200 ALC/μL. Participants who were nucleocapsid antibody positive had serologic evidence of SARS‐CoV‐2 exposure. A linear model regressed log2 SARS‐CoV‐2 spike RBD antibody levels on vaccine type (including none) and log2 ALC; age, body mass index, and sex were controlled for in the model. This analysis set included 174/179 unvaccinated and vaccinated nucleocapsid positive participants (five time points were missing for two participants who received an mRNA vaccination and three who were unvaccinated). ALC, absolute lymphocyte count; RBD, receptor binding domain; SARS‐CoV‐2, severe acute respiratory syndrome coronavirus 2.

There was no evidence of an association between participant characteristics and spike RBD antibody levels in those with serologic evidence of SARS‐CoV‐2 exposure. In 174 participants with serologic evidence of SARS‐CoV‐2 exposure and ALC samples available, ALC was not predictive of spike RBD antibody levels, regardless of vaccination type or status (Fig. 7).

### Safety

A total of 10% (15/148) of vaccinated participants contracted COVID‐19 after being fully vaccinated (confirmed = 12; suspected = 3); all events were nonserious and not severe. Thirty‐three percent (5/15) of participants who contracted COVID‐19 after being fully vaccinated had spike RBD antibody results available, corresponding to a mean (range) of 4936 (76.2–11,440) U/mL. In the 77.4% (103/133) of fully vaccinated participants who did not contract COVID‐19 and had spike RBD antibody results available, mean (range) spike RBD antibody levels were 846 (0.4–44,260) U/mL. Eleven of the COVID‐19 cases were considered moderate and four were mild. Ozanimod treatment was continued in nine participants and temporarily interrupted in five (one unknown). Eleven participants recovered by the time of data cutoff, and one recovered with sequelae (cough and loss of sense of smell). All participants subsequently recovered.

## Discussion

The results of this retrospective analysis of the DAYBREAK open‐label extension trial demonstrated that most participants with RMS receiving long‐term treatment with ozanimod mount a serologic response to SARS‐CoV‐2 vaccination and infection based on measurement of spike RBD antibody levels. In this analysis, all COVID‐19–related AEs post–full vaccination in participants taking ozanimod were nonserious and not severe.

While most ozanimod‐treated participants in this study had postvaccination spike RBD antibody levels that met the criteria for seroconversion, heterogeneity in the magnitude of serologic response was observed, with some participants developing low (<0.8 U/mL; seroconversion cut‐off) antibody levels. This finding is not unexpected because varying levels of spike RBD antibodies were reported in the general population,^26^ including homogenous populations of healthy individuals.^27^ In addition, a large, real‐world study using the Siemens Healthineers Atellica IM SARS‐CoV‐2 IgG assay and different sample timing relative to vaccination compared with the analysis presented here, plus no assessment of previous SARS‐CoV‐2 exposure, showed S1P receptor modulators (primarily fingolimod) to be similar to anti‐CD20 therapies with regard to patients with MS achieving lower serologic responses compared with other DMTs.^28^ These findings highlight the complexity of postvaccination serologic responses across DMTs.^28^ Given the heterogeneity of antibody response, some patients with RMS who are taking S1P receptor modulators and other immunomodulating therapies may benefit from mitigation strategies (e.g., booster doses^29^, ^30^, ^31^) beyond the primary SARS‐CoV‐2 vaccination series to allow for development of a more robust, postvaccination immune response.^29^, ^30^, ^31^, ^32^

Predictors of serologic response to vaccination would be useful when developing tailored treatment approaches for patients with MS taking ozanimod; however, no association between age, ALC, BMI, or sex and spike RBD antibody levels or seroconversion was found in the present analysis. These findings align with other studies that also reported no association between seroconversion after vaccination against SARS‐CoV‐2 and the demographic or clinical characteristics of patients with MS^7^, ^10^, ^33^; however, there are conflicting data in the literature regarding associations between ALC and RBD antibody levels.^10^, ^32^, ^33^, ^34^, ^35^ Additional studies are needed to evaluate other patient characteristics as predictors of serologic response given these conflicting data. In the analysis presented here, vaccination type was the only predictor of spike RBD antibody levels and seroconversion, where mRNA vaccination was predictive of higher spike RBD antibody levels and seroconversion rates compared with non‐mRNA vaccines.

The high rate (100%) of seroconversion in ozanimod‐treated participants with RMS after SARS‐CoV‐2 mRNA vaccination contrasts with data that observed failure to develop a postvaccination humoral response in fingolimod‐treated patients with MS fully vaccinated against SARS‐CoV‐2 with the Pfizer vaccine (determined with EUROIMMUN anti‐SARS‐CoV‐2 IgG quantitative ELISA assay/AGILITY ELISA analyzer [DYNEX]).^8^ Although the number of studies reporting the impact of S1P receptor modulators on SARS‐CoV‐2 vaccination is relatively small at this time, data currently suggest that seroconversion rates are generally higher in patients treated with ozanimod (84%–100%) than fingolimod (28%–77%).^15^, ^21^, ^32^, ^36^, ^37^, ^38^ Differences in seroconversion rates between fingolimod and ozanimod may be due to variations in the assays used between studies or may be a result of differences in S1P receptor specificity, effects on influencing the adaptive immune system, or patient backgrounds with respect to prior treatment exposures or other host factors.^38^, ^39^ In addition, assays vary in terms of sensitivity to detect prior infection and in the durability of measured responses, leading to discrepancies between data sets.^40^

In ozanimod‐treated participants who received an mRNA vaccine, the spike RBD antibody levels observed in this analysis ranged from approximately 1.3 U/mL (in participants with no serologic evidence of SARS‐CoV‐2 exposure) to 44,260 U/mL (in participants with serologic evidence of SARS‐CoV‐2 exposure). Using the Elecsys assay implemented in this study, Sormani et al reported post‐SARS‐CoV‐2 mRNA vaccine antibody levels to range from approximately 100 U/mL to 10,000 U/mL in untreated patients with MS; 100 U/mL to 100,000 U/mL in patients taking glatiramer acetate, interferon, alemtuzumab, cladribine, dimethyl fumarate, teriflunomide, or natalizumab; and from 1 U/mL to 31,623 U/mL in patients taking rituximab, fingolimod, or ocrelizumab.^10^ However, the serum samples assessed in Sormani et al were consistently 4 weeks postvaccination, whereas the timeframe of our analysis ranged broadly from 1 day to 229 days (median of 60 days), which may have affected the measurement of immune response because a number of samples were acquired very shortly after vaccination or after spike RBD antibody levels had decreased from their peak response. In general, contrasting immune response data to vaccination with that of other studies must be done with discretion, because large heterogeneity was observed across commercial assays in terms of antibody responses to SARS‐CoV‐2 and inherent problems with cross trial comparisons.^40^ Finally, although manufacturers of SARS‐CoV‐2 antibody assays generally have predefined spike antibody levels considered to represent seroconversion, a consensus on protective spike RBD antibody index values is not established, making interpretation of these comparisons additionally challenging.

All COVID‐19–related AEs post–full vaccination in participants taking ozanimod were nonserious and not severe in our analysis. These findings suggest that SARS‐CoV‐2 vaccination is effective in limiting disease course in ozanimod‐treated participants with RMS who contracted COVID‐19; however, larger population sizes are needed to validate these observations.

This study has other limitations. This retrospective analysis leveraged data acquired from an ongoing clinical trial designed to study the long‐term safety and efficacy of ozanimod and was not intended to assess the impact of SARS‐CoV‐2 vaccinations or COVID‐19 outcomes in study participants. As such, all biological samples were convenience samples obtained per protocol for safety assessments, and the timing of serum collection was not optimized to determine serologic responses to SARS‐CoV‐2 vaccination. Further, the biological samples acquired did not include peripheral blood mononuclear cells, so cellular responses to SARS‐CoV‐2 vaccination could not be assayed. Lastly, determination of SARS‐CoV‐2 exposure was limited to nucleocapsid antibody testing which may have caused some selection bias, and because all participants in this study were treated with ozanimod, a reference population of participants who were untreated or treated with other therapies was not available for comparison.

In conclusion, these data suggest that most ozanimod‐treated participants with RMS mount a serologic response to SARS‐CoV‐2 infection and SARS‐CoV‐2 vaccination, with a more robust response to mRNA vaccines than non‐mRNA vaccines, regardless of participant characteristics or ALC levels, thereby supporting the use of SARS‐CoV‐2 vaccines for most ozanimod‐treated participants with RMS.

## Author Contributions

Conception and design of the study: AK, BACC, JKS, RM, SH. Acquisition and analysis of data: AK, DS, EB, JKS, RM, SH, YH, YL. Data interpretation: all authors. Drafting a significant portion of the manuscript or figures: all authors.

## Conflict of Interest

BACC reports personal compensation for consulting for Alexion, Atara, Autobahn, Avotres, Biogen, Boston Pharma, EMD Serono, Gossamer Bio, Horizon Therapeutics, Immunic AG, Neuron23, Novartis, Sanofi, TG Therapeutics, and Therini, and received research support from Genentech. RM, AK, EB, YI, YH JKD, DS, and SH are employees and/or shareholders in Bristol Myers Squibb. ABO has received fees for advisory board participation and/or consulting from Accure, Atara Biotherapeutics, Biogen, BMS/Celgene/Receptos, GlaxoSmithKline, Gossamer, Janssen/Actelion, MedImmune, Merck/EMD Serono, Novartis, Roche/Genentech, and Sanofi‐Genzyme; and has received grant support to the University of Pennsylvania from Biogen Idec, Merck/EMD Serono, Novartis, and Roche/Genentech. HPH reports personal fees for consulting, serving on steering committees, and speaking from Bayer Healthcare, Biogen, Celgene, GeNeuro, Genzyme, MedImmune, Merck, Novartis, Octapharma, Roche, Sanofi, and Teva.

## Data Availability

BMS policy on data sharing may be found at https://www.bms.com/researchers‐and‐partners/independent‐research/data‐sharing‐request‐process.html.
